# Innate lymphoid cells: More than just immune cells

**DOI:** 10.3389/fimmu.2022.1033904

**Published:** 2022-10-26

**Authors:** Le Xiong, Stephen L. Nutt, Cyril Seillet

**Affiliations:** ^1^ Immunology Division, Walter and Eliza Hall Institute of Medical Research, Melbourne, VIC, Australia; ^2^ Department of Medical Biology, University of Melbourne, Melbourne, VIC, Australia

**Keywords:** neuroimmune interaction, IL-22, ILC3, ILC2, ILC1, physiological sensors, metabolic homeostasis, gut homeostasis

## Abstract

Since their discovery, innate lymphoid cells (ILCs) have been described as the innate counterpart of the T cells. Indeed, ILCs and T cells share many features including their common progenitors, transcriptional regulation, and effector cytokine secretion. Several studies have shown complementary and redundant roles for ILCs and T cells, leaving open questions regarding why these cells would have been evolutionarily conserved. It has become apparent in the last decade that ILCs, and rare immune cells more generally, that reside in non-lymphoid tissue have non-canonical functions for immune cells that contribute to tissue homeostasis and function. Viewed through this lens, ILCs would not be just the innate counterpart of T cells, but instead act as a link between sensory cells that monitor any changes in the environment that are not necessarily pathogenic and instruct effector cells that act to maintain body homeostasis. As these non-canonical functions of immune cells are operating in absence of pathogenic signals, it opens great avenues of research for immunologists that they now need to identify the physiological cues that regulate these cells and how the process confers a finer level of control and a greater flexibility that enables the organism to adapt to changing environmental conditions. In the review, we highlight how ILCs participate in the physiologic function of the tissue in which they reside and how physiological cues, in particular neural inputs control their homeostatic activity.

## Introduction

Non-cytotoxic ILCs were discovered in early 2010’s and have been considered as innate counterpart of T lymphocytes, constituting an early source of cytokines during infection while the adaptive response can be mounted and eradicate the pathogens. However, ILCs possess singular features that make them stand out of the immune landscape. Firstly, they are relatively rare in lymphoid tissues and in the circulation but reside in non-lymphoid organs and particularly in mucosal surfaces (1). Secondly, ILCs can develop in absence of recombination-activating genes, Rag-1 and Rag-2, and therefore do not express the antigen receptors characteristic of T and B lymphocytes. They are recognized as part of innate immunity but are not devoid of memory responses (2–5). Finally, while other innate immune cells rely on pattern-recognition receptors (PRRs) to initiate their activation, the role of PRR such as Toll-like receptors (TLR) in ILCs remains unclear (6, 7). ILCs are equipped with a plethora of receptors that can sense host-derived signals such as dietary metabolites, microbial products, hormones, neuropeptides, and cytokines. Although, ILCs are involved in the immune protection against pathogens and noxious stimuli, accumulating evidence also shows that the constitutive activities of ILCs contribute to maintaining homeostasis of organs where they reside.

The homeostatic processes that maintain the physiological functions of each tissue involves three main components, a sensor, a control center, and an effector. The sensors detect information from the changing environment, the control center processes the information and transmit appropriate responses to the effector. In this hierarchy, ILCs function within the control center to modulate the activity of effector cells by integrating host-derived signals. In contrast to pathogenic cues that will skew the immune responses depending on the pathogens, these physiologic modulators balance the type 1, 2 and 3 response depending on the tissue. A lot of work has been done to elucidate the wide range of mediators that ILCs can sense and produce; however, the extent of the regulatory network in which ILCs are involved remains underappreciated. ILCs can secrete not only cytokines but also hormones, neurotransmitters, or growth factors. In turn, ILCs are also equipped to sense these mediators and can therefore create a bidirectional communication with other sensory and effector cells in the environment. Probably the most dynamic and reciprocal communication is the neural immune interactions that have been described in mucosal tissues. The nervous and immune system share so many functional and molecular properties that it is tempting to consider the neuroimmune system as whole. Indeed, they are both widespread throughout the organism, composed of a large diversity of cells, and rely on an intense network of communication. Immunologists are now appreciating that both systems share similar messengers as neurons can sense and secrete cytokines such as IL1-β (8), IL-6 (9), TNF-α (10), or TGF-β (11) which can modulate cognitive functions (12). Sensory and effector capacity of both systems is critical to monitor environmental changes and instruct adequate responses.

In this review, we highlight how ILCs participate in the tissue functions and integrate nonpathogenic signals to maintain homeostasis. The latest studies on the homeostatic functions of ILCs contribute to the understanding of their evolutionary conservation. If early studies have shown functional redundancy between ILCs and adaptive lymphocytes during pathogen infection, it becomes apparent that their uniqueness shines in physiology. The organism uses ILCs to modulate type 1, 2 and 3 responses in sterile condition to adapt tissue physiology to non-pathogenic perturbation of the homeostasis, such as fasting, cold exposure or chronic stress. These perturbations require types of responses that cannot be driven by antigen-specific T cells.

This concept could represent an important step in our understanding of how sterile inflammation can emerge and ultimately lead to chronic inflammation and disease.

## The innate lymphoid cell family

ILCs have been divided into five subsets. Group 1 ILCs (ILC1s) include two subsets of T-bet expressing ILCs composed of NK cells and type 1 ILCs. They participate to type 1 immune responses by producing IFN-γ. NK cells are considered as cytotoxic ILCs and circulate in the blood or lymphatic system to wipe out tumor cells or viral invaders, with the aid of different surface activating or inhibitory receptors and potent cytotoxicity. In contrast, ILC1s are resident in tissues like liver, adipose tissues, intestines, and salivary gland and can be activated by soluble cytokines such as IL-15, IL-12, and IL-18. ILC1s exert rapid and first-line responses to protect host from the infection of viruses and intracellular bacteria at the initial site of invasion through producing effector cytokines (13).

Group 2 ILCs (ILC2s) are characterized by the expression of GATA-binding protein 3 (GATA3) and are resident in mucosal tissues such as the lungs, gastrointestinal tract, tonsil, and skin (14). They are early effectors in type 2 immune responses, releasing cytokines like IL-5, IL-13, IL-4, and epidermal growth factor family member amphiregulin to fight against helminths and regulate tissue repair (14).

Group 3 ILCs (ILC3s) are defined by RORγt expression, similar to Th17 cells. Based on surface markers, ILC3s are divided into NKp46^+^ ILC3 and NKp46^–^ ILC3 and produce effector cytokines including IL-22, IL-17, GM-CSF, IFN-γ, TNF-α as well as growth factor HB-EGF (heparin-binding epidermal growth factor–like growth factor) (15, 16). ILC3s are abundant at intestinal mucosa, skin, lungs, and mesenteric lymph nodes, generating rapid immune responses against extracellular microbes and regulating tissue homeostasis (17). The final ILC subset is the lymphoid tissue-inducer cells that are also dependent on RORγt and are derived from a fetal liver progenitor. They are critical to orchestrate secondary lymphoid organogenesis during embryogenesis (18, 19).

## Metabolic and thermal homeostasis: Adipose tissue

Adipose tissues, composed of white adipose tissue (WAT), beige adipose tissue, and brown adipose tissue, serve as an energy reservoir and mediate energy expenditure by regulating lipolysis, insulin sensitivity and thermogenesis. Adipose tissue ILCs, especially ILC1 and ILC2, have emerged as key immune cells in both physiological and obese conditions.

**Figure 1:**
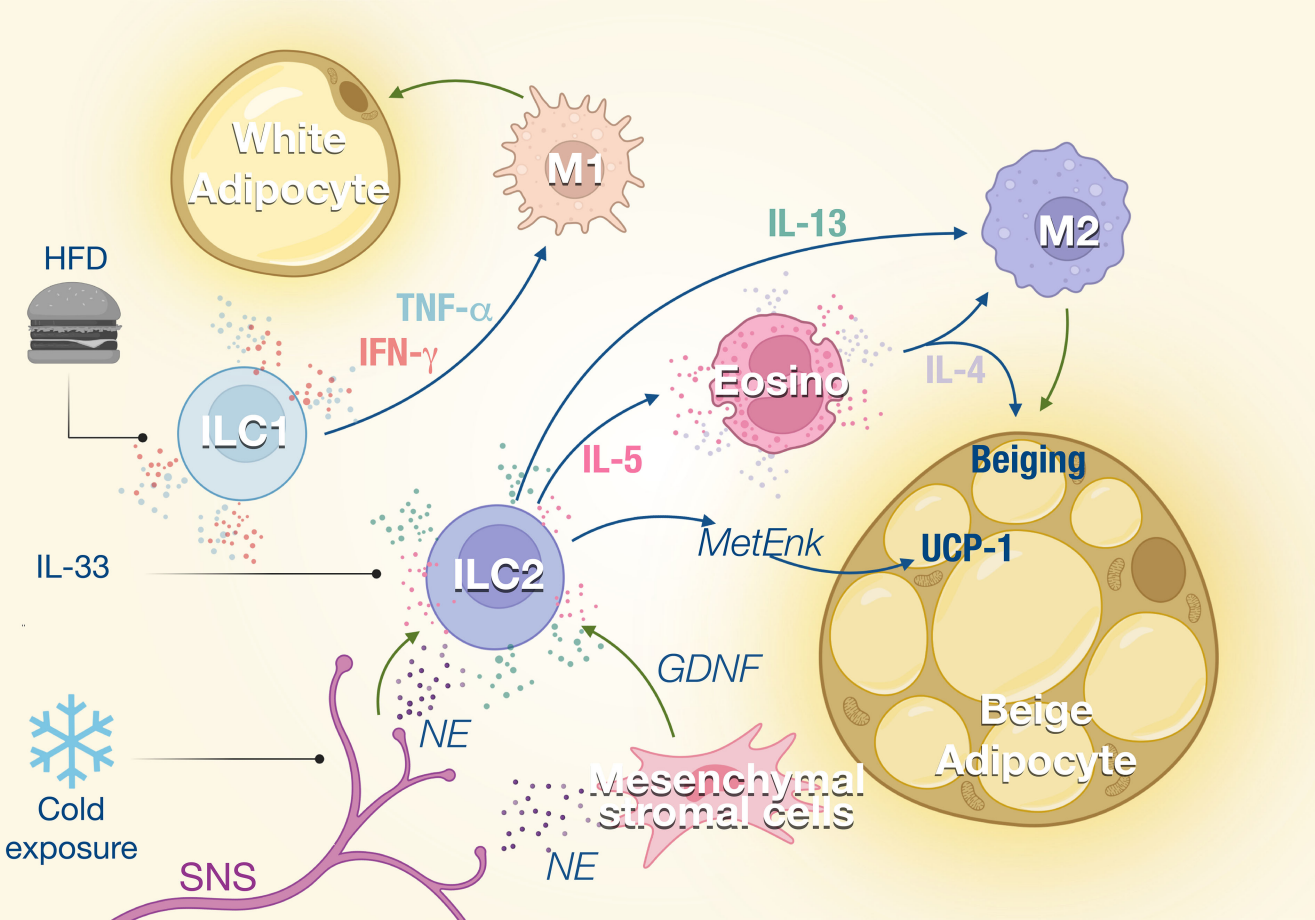
ILCs in the adipose tissue. ILC1s and ILC2s regulate the balance between M1 and M2 macrophages in the adipose tissue. External stimuli such as high-fat diet (HFD) or cold exposure modulate the activity of the ILC which impacts the M1/M2 ratio in the tissue. ILC1 promotes M1 macrophage polarization through the production of TNF-α and IFN-γ, which increases not only lipid storage but also inflammation and adipose fibrosis. ILC2s are activated by IL-33 and regulated by the sympathetic nervous system (SNS) in response to cold exposure. Consequently, IL-5 and IL-13 production by ILC2s is enhanced, which promotes eosinophil recruitment and M2 macrophage polarization, respectively. This type 2 response promotes the beiging of adipose tissues and thermogenesis.

The metabolic homeostasis and insulin sensitivity depend on the balance between type 1 and type 2 immune response. During metabolic disorder and insulin resistance, the type 1 inflammation is increased with the expansion of inflammatory macrophages (M1) while type 2 and anti-inflammatory macrophages (M2) are inhibited. M2 macrophages are essential to support the anti-inflammatory microenvironment in the adipose tissue and maintain metabolic fitness. ILC1 and NK cells which promote type 1 inflammation are reported to induce local inflammation of adipose tissues during obesity (20). At steady state, adipose NK cells and ILC1 show a natural cytotoxicity against adipose macrophages which limit their expansion. Upon high-fat diet (HFD) feeding, the cytotoxicity of NK cells and ILC1 is reduced which contributes to the accumulation of M1 macrophages (21). HFD also induces the production of IL-12 which stimulates the proliferation and the activation of NK cells and ILC1 in adipose tissue (20) (**Figure 1**). NK cells and ILC1 from HFD mice produce IFN-γ which promotes M1 macrophage polarization and leads to insulin resistance (20). The proportion of IFN-γ^+^ NK cells and ILC1 is increased in omental adipose of obese individuals and is even higher in obese patients developing type 2 diabetes. Adipose IFN-γ^+^ NK cells correlate with elevated blood glucose levels, while adipose tissue ILC1s are positively associated with body mass index and insulin resistance (22, 23). Insulin resistance and diabetes are directly linked to tissue fibrosis that is induced by M1 macrophages in response to transforming growth factor β-1 (TGF-β1) signaling (22). ILC1 from obese and obese diabetic patients produce higher IFN-γ and induce the expression of fibrosis-related genes in macrophages (23). Interestingly, mice under HFD show increased adipose fibrosis and more severe glycemic intolerance after the transfer of adipose ILC1 from previously HFD-fed mice compared to those that did not (23). Therefore, ILC1s participate to metabolic fitness through the constitutive cytotoxicity toward adipose macrophages and control of the M1 and M2 balance. HFD disturbs ILC1 function, which skews adipose tissue towards a pathogenic accumulation of pro-inflammatory macrophages and promotion of tissue fibrosis (**Figure 1**).

A type 2 immune response is important in maintaining homeostasis of lean adipose tissues and regulating thermogenesis. ILC2s are central player in type 2 immunity as a source of IL-5 that supports eosinophil expansion and survival, which in turn induces alternative activated macrophages M2 (24, 25) (**Figure 1**). ILC2s also modulate non-immune compartments and contribute with eosinophil-derived IL-4, to support the expansion and lineage commitment into beige adipocytes from adipocyte precursors (26).

ILC2s also regulate core body temperature in response to cold challenge by inducing adaptive thermogenesis through beiging of white adipose tissue (24, 25). Beige adipose tissue is critical for the healthy function of adipose tissues through the droplet formation and fatty acid uptake and thermal heat (27). IL-5 and MetEnk (Methionine-enkephalin) peptides endogenously produced by ILC2 increase the expression of thermogenic gene UCP1 (uncoupling protein-1) in beige adipocytes and thermogenesis in WAT (24, 28). With aging, thermogenesis becomes less efficient and recently Goldberg et al. demonstrated that ILC2 were gradually lost with aging in the adipose tissue (29, 30). Interestingly, the proportion of ILC2 was retained in old mice when they were maintained on calorie-restricted diet, consistent with several studies that observed that ILC2s in WAT decline in obese human and mice (24, 31, 32). The decline of ILC2s correlates with a loss of eosinophils in the adipose tissue and an impaired thermogenic response after cold challenge in aging mice that failed to induce UCP1. Adoptive transfer of ILC2s from young adult mice protected old mice from cold challenge indicating that aged ILC2s are intrinsically defective (29). The roles of ILC2 in thermoregulation have also been illustrated in the skin where the TRPM8^+^ neurons which sense environmental cold stimuli, activate the skin ILC2s *via* IL-18 signaling, resulting in upregulated expression of UCP1 in dermal cells and increased thermogenesis (28).

Sympathetic nerves also control ILC2 function during homeostasis and in response to stress. Cold exposure induces the secretion of catecholamines (33), which can be sensed by ILC2s which express β-adrenergic receptors (34). Chemical sympathectomy by 6-hydroxydopamine (6-OHDA) leads to a reduction of the ILC2s and eosinophils in the adipose tissue, which is correlated with a significant decrease of UCP1 (35). Sympathetic innervation also controls IL-5 and IL-13 expression by ILC2s through the secretion of GDNF (glial-derived neurotrophic factor) by mesenchymal stromal cells (36). Disruption of the neuroimmune interaction led to increased susceptibility to obesity and insulin resistance (36). These effects of the SNS on the ILC2 function could also contribute to explain the weight gain observed with the use of β-blockers.

The balance between the type 1 and 2 response is critical for the metabolic homeostasis. The constitutive activity of the ILC2 has been shown to be pivotal. Understanding how the basal activity of these cells is regulated by host derived mediators is important to better appreciate the link between environmental changes and their consequences on the ILC activity in the adipose tissue. The study of the neuroimmune interactions in the adipose tissue could also lead to several opportunities to repurpose the wide array of neuromodulators currently used in medical therapy for novel and previously unanticipated indications to treat obesity and metabolic disorders.

## Tissue repair and regeneration: The lungs

### Regulation eosinophil migration

**Figure 2:**
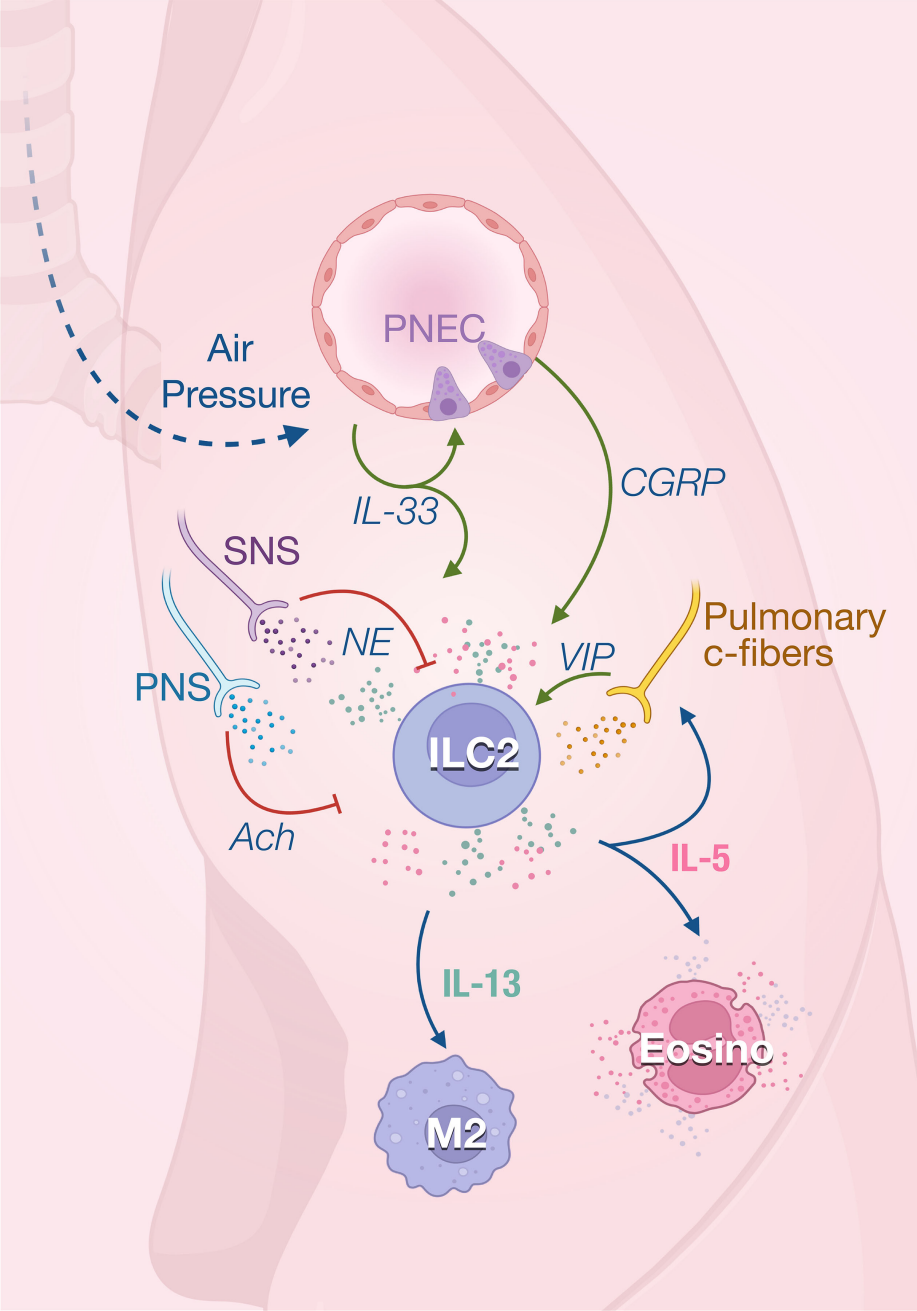
ILC2 in the in lungs. ILC2 activity is rapidly induced by the first breath which promotes IL-33 secretion in the lungs. IL-33 acts on ILC2s to induce their production of both IL-5 and IL-13 that regulate the homeostasis of eosinophils and M2 macrophages. IL-33 also promotes ILC2 activity through the secretion of calcitonin gene‐related peptide (CGRP) produced by pulmonary neuroendocrine cells (PNECs). IL-5 derived from ILC2s, which are stimulated by VIP, further promotes vasoactive intestinal peptide (VIP) production by pulmonary c-fibers, creating a positive feedback loop. The parasympathetic nervous system (PNS) and SNS mainly act as negative feedback loop in response to inflammatory signals and prevent tissue damage.

The anti-inflammatory properties of type 2 immune responses observed in adipose tissue are also important for maintaining lung homeostasis. At steady state, ILC2 is the predominant subset resident in the lungs. ILC2s appear in the lungs early from perinatal stage to rapidly expand after birth and are located in collagen-rich areas near medium-sized blood vessels (37, 38). ILC2s contribute to lung quiescence in homeostasis by fostering macrophage polarization towards a M2 phenotype as they are the unique source of IL-13 at steady state in the lungs (39). First breathing in the alveolar space and abrupt change in pressure rapidly increase IL-33 expression in lungs, inducing the expansion of ILC2s and their production of IL-13 promotes M2 polarization (39, 40) (**Figure 2**). ILC2s also constitutively express IL-5 which has been shown to regulate the eosinophils homeostasis (38) while the absence of ILC2 leads to a drastic loss of circulating eosinophils (41).

The lungs are innervated with peripheral sensory neurons which are derived from vagal and spinal sensory nerves. Most sensory neurons express nociceptors, initiating reactions like cough and bronchoconstriction after sensing different types of airway stimuli. The vasoactive intestinal peptide (VIP) has been shown to stimulate IL-5 production in pulmonary and intestinal ILC2s (38). IL-5 secretion by lung and gut ILC2s shows a strong circadian rhythm with a nadir during nighttime in rodents correlating with their feeding behavior (42). Food intake stimulates the secretion of VIP that is sensed by VIPR2 on ILC2 which creates oscillating production of IL-5 which synchronized the eosinophils numbers with metabolic cycling (38). IL-5 in turn directly acts on nociceptors to trigger the secretion of VIP which subsequently induces more IL-5 production *via* VIP-VIPR2 signaling pathway. The positive neuro-immune loop amplifies type 2 inflammation mediated by ILC2s (43). The homeostatic role of eosinophils is unclear, however recent studies have report new physiological functions for these cells in metabolic control and lipid absorption (44, 45), control of inflammation (46, 47) and gut motility (45). It is therefore important to understand how ILCs integrate environmental cues to modulate the eosinophils functions at steady state.

### Neural protection of tissue injury

To maintain the tissue homeostasis, it is critical to control inflammation and promote tissue repair. The failure to initiate tissue regeneration results in chronic inflammation and fibrosis. In addition to critical type 2 effector functions, ILC2s play important roles in direct and indirect promotion of wound healing and tissue repair. During type 2 inflammation like *Nippostrongylus brasiliensis* (helminth) infection or asthma, ILC2s co-express IL-5 and IL-13, inducing stroma production of eotaxin (chemokine) and eosinophil accumulation (38, 48). Neural interactions with ILC2 in the tissue have been identified to inhibit ILC2 inflammatory functions and protect from tissue damage. The lungs are innervated with both parasympathetic and sympathetic nervous systems which play essential roles in regulating respiratory functions and pulmonary homeostasis.

### Parasympathetic and sympathetic nervous systems

The parasympathetic nervous system (PNS) delivers cholinergic contractile innervation and mediates bronchoconstriction in the lung through the secretion of acetylcholine (ACh). ACh induces the cholinergic signals in target cells *via* two types of receptors, the nicotinic ACh receptors (nAChRs) and the muscarinic ACh receptors (mAChRs). ILC3s are an additional source of acetylcholine in allergic airway inflammation (49). Lung ILC2s constitutively express α7nAChR which can be upregulated by IL-25 or IL-33 (50). Stimulation of the α7nAChR with a specific agonist attenuates GATA3 expression, proliferation and the production of IL-5 and IL-13 by ILC2s. Therefore, the engagement of α7nAChR inhibits airway inflammation induced by IL-33 or *Alternaria Alternata* infection and restores lung functions (51). Genetic deletion of *Chrna7* which encodes α7nAChR increases the number of ILC2s in the lung and worsens allergic reactions, suggesting an inhibitory effect of ACh-α7nAChR signaling on ILC2 responses (52). Cholinergic regulation of ILC2 is bidirectional as ILC2s express choline acetyltransferase (ChAT) and can therefore produce ACh. While some ILC2s constitutively produce ACh, it is strongly upregulated during parasitic infection and allergic reaction (53, 54). Specific deletion of ChAT in ILC2s limits their proliferation and capacity to produce IL-5 and IL-13, leading to impaired immunity against *Nippostrongylus brasiliensis* infection. Interestingly, the expression of ChAT is maintained over three weeks after helminth eradication, suggesting that this sustained expression could mediate the tissue repair function of ILC2 (54). The apparent opposite effect of ACh on the ILC2 function could be attributed to the different receptors that they express. ILC2 express both muscarinic and nicotinic receptors, but their expression is modulated by inflammatory signals such as IL-25 or IL-33. Hence, depending on the receptor engaged, it is possible that the cholinergic signaling fosters the pro-inflammatory functions of the ILC2 early in the infection but has negative regulation during the resolution phase.

The sympathetic nervous system (SNS) innervating the lungs which stimulates bronchodilation and mucus production also appears to negatively modulate ILC2 during inflammation and prevent tissue damage. Human and murine lung ILC2s express high levels of β_2_AR. ILC2s lacking β_2_AR excessively proliferate and produce type 2 cytokines in the lungs following helminth infection, suggesting a regulatory role of sympathetic pathway in type 2 inflammation (34).

### CGRP (calcitonin gene‐related peptide)

Most of the afferent nerve fibers innervating the respiratory tract are thin unmyelinated fibers, called C-fibers (55), that are quiescent in healthy lungs but can be activated by inflammation. These sensory fibers express nociceptors transient receptor potential (TRP) channels TRPV1 and prostaglandin E2 receptor (56) which results in increased secretion of neuropeptides like VIP, substance P and calcitonin gene-related peptide (CGRP). Activation of bronchopulmonary C-fibers with capsaicin, a selective TRPV1 agonist, increases allergic airway inflammation, while nociceptor silencing reduces inflammation (43). A bidirectional communication has been described between these nociceptors and ILC2. In response to inflammation, IL-5 secreted by ILC2 directly activates pulmonary C-fibers that will produce VIP. VIP increases ILC2 activation and induces Th2 recruitment, leading to excessive lung inflammation (43).

Pulmonary C-fibers and pulmonary neuroendocrine cells (PNECs) express CGRP which also regulates ILC2s in the lungs. Under steady condition, lung ILC2s express CGRP receptor subunits Ramp1 and Calcrl, and nearly 20% of pulmonary ST2^+^ILC2s can produce CGRP (57, 58). Therefore, CGRP can regulate ILC2 activity in a paracrine or autocrine manner. ILC2s are found near PNEC in airway branch points (59). As a key second messenger of CGRP, cyclic AMP mediates the effects of CGRP on IL-33-activated ILC2. Cell-permeable dibutyryl-cAMP stimulation similarly inhibited ILC2 proliferation and enhanced IL-5 production induced by IL-33 (57, 58, 60). Upon inflammation, PNECs secrete CGRP which stimulates IL-5 production by ILC2s. Conversely, deletion of the CGRP receptor on ILC2s reduces the type 2 immune responses to allergens (59). Interestingly, incubation of ILC2 with CGRP *in vitro* could rapidly promote their expression of IL-5 and amphiregulin within 6 hours. However, after 3 days of culture in presence of IL-33, the production of IL-5 and IL-13 are inhibited while there is still increased amphiregulin (57). In this study, CGRP inhibits eosinophilia and hypersensitivity through suppressing ILC2 proliferation and secretion of IL-5 and IL-13 following *in vivo* administration of IL-33 (57). This indicates that CGRP, as was the case for ACh, can have different modulatory effects depending on the time and activation status of ILC2s. It is also possible that neuropeptides target different ILC2 subsets depending on their expression of neuroreceptors. Indeed, inflammatory ILC2s that are found in the lung and intestine characterized by their high expression of KLRG1 (61) are less responsive to CGRP than lung resident natural (ST2^+^) ILC2 (57).

## Exchange with environment and maintenance of barrier integrity: The intestine

As one of the largest barriers confronting the external environment, the gut is exposed to a diverse and symbiotic microbiome, different microbial pathogens, and diets, and is also home to host immune cells, hormones, and neuronal complexes (62). From lumen to mesentery, the intestine is structurally composed of epithelium, lamina propria, submucosa, muscularis, and serosa. In homeostatic conditions, intestinal immune cells respond to non-pathogenic stimuli including metabolites, microbial peptides, hormones, and neuropeptides. These molecules regulate the activity of immune cells which in turn modulate neurons and epithelial cells to maintain intestinal function and barrier integrity.

Gut epithelium mainly contains T cells, while different types of immune cells involving macrophages, dendritic cells, ILCs, granulocytes, T cells, and B cells dwell in the lamina propria (63). Of these immune cells, resident ILCs appear to act as a central sensor integrating various signals to sustain mucosal homeostasis (64, 65).

### Intestinal ILCs

**Figure 3:**
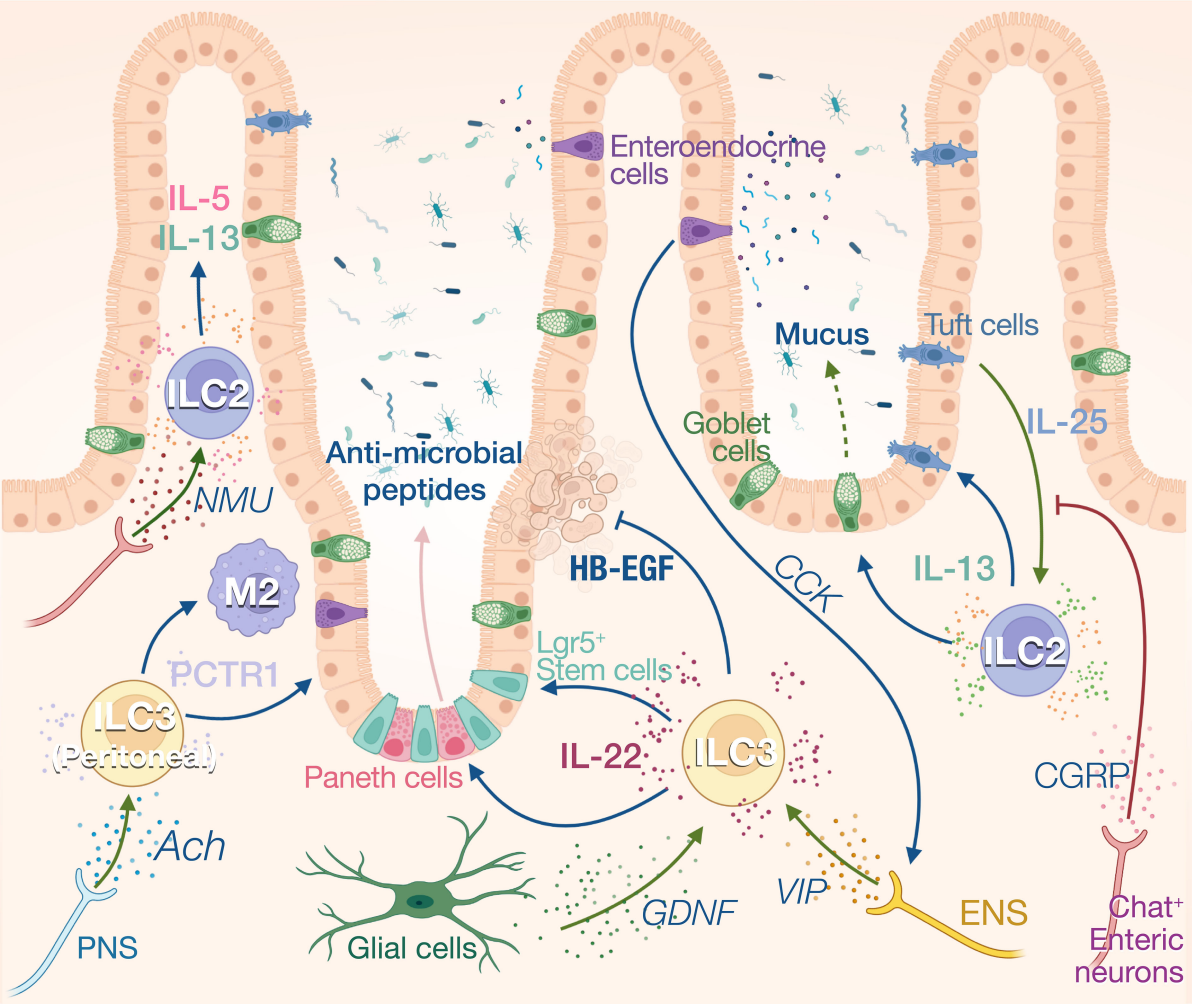
Neuro-ILCs interaction in intestinal tract and peritoneal tissue. Intense communications between ILCs and nerves have been described in the gut. Constitutive expression of IL-25 by Tuft cells regulates ILC2 numbers and IL-13 expression. IL-13 in turn fosters Tuft cell differentiation and induces mucus production by goblet cells. Activation of ILC2s by IL-25 is inhibited by CGRP produced by ChAT^+^ enteric neurons. These neurons can also express NMU which activates ILC2s during inflammation. ILC3s are the main producers of IL-22 at steady state which regulates the anti-microbial production by Paneth cells and the proliferation of intestinal stem cells. Heparin-binding epidermal growth factor–like growth factor (HB-EGF) produced by ILC3 protects intestinal epithelial cells from TNF-induced cell death. IL-22 production is enhanced by enteric nervous system (ENS)-derived VIP in response to cholecystokinin (CCK) secreted by enteroendocrine cells that are activated by food intake. ILC3s also respond to glial-derived neurotrophic factor (GDNF) produced by enteric glial cells. Peritoneal ILC3s are regulated by the PNS through the neurotransmitter acetylcholine (ACh). Specifically, ACh promotes the production of protectin conjugates in tissue regeneration 1 (PCTR1) by peritoneal ILC3s, enhancing tissue resolution after inflammation.

ILC3s reside in small intestine and colon lamina propria and are the main constitutive source of IL-22 (66). IL-1β and IL-23 from myeloid cells activate ILC3s which increase their secretion of IL-22, IL-17, and GM-CSF (67). IL-22 is a critical modulator for intestinal mucosa through binding to IL-22 receptors on intestinal epithelial cells (68). At steady state, IL-22 limits commensal bacteria from entry into the gut by eliciting the synthesis of antimicrobial peptides (AMPs) involving RegIIIβ, RegIIIγ, S100A, and S100B (69–71) (**Figure 3**). IL-22 also promotes epithelial expression of fucosyltransferase 2 (Fut2) and fucosylation, which contributes to the resistance and tolerance to pathogenic bacteria (72, 73). Fut2^+^ Paneth cells also contribute to antimicrobial defenses by secreting α-defensin from granules (74). N-glycosylation mediated by IL-22 encourages the expansion of commensal bacteria that compete with *Clostridioides difficile* for the dietary niche (75). During pathogen infection, IL-22 also drives physical expulsion of helminths by driving mucin production and goblet cell hyperplasia or bacteria by increasing claudin-2-mediated tight junction permeability and consequent diarrhea (76–78). However, uncontrolled IL-22 production by ILC3 can be deleterious for the host, contributing to dysbiosis and promoting pathogen expansion (79). Indeed, *Salmonella Typhimurium* exploits ILC3 to produce IL-22 to cause host dysbiosis, thus facilitating its colonization through competition with the gut commensal microbiota (80). To restrain bacteria growth, ILC3 number is limited by pyroprosis which reduces the IL-22 levels, representing a potential host defense mechanism (80). ILC3s not only regulate gut microbial homeostasis through epithelial cells but also present antigens to induce Rorγt^+^ Treg cell differentiation, and produce IL-2 to support their maintenance in the gastrointestinal tract, thus contributing to the maintenance of specific tolerance against the microbiota (81–83).

In addition to keeping microbiota symbiosis, ILC3 also contributes to the maintenance of intestinal barrier integrity and promote tissue repair. IL-22 activates and preserves the proliferation of Lgr5^+^intestinal stem cells (ISCs) after tissue damage to sustain epithelial regeneration (84, 85). HB-EGF, predominantly secreted by ILC3, protects intestinal epithelial cells from TNF-induced cell deaths and experimental colitis (86). Contradictory findings suggest that IL-22 has either pro- or anti- carcinogenic effects during epithelial regeneration. Effective DNA damage recovery following genotoxic stress can be initiated by IL-22, whereas this cytokine potentially promotes tumor development during epithelial recovery stage (87, 88). After tissue injury, ILC3s can also directly sense cellular damage resulted from neutrophil apoptosis which releases lysophosphatidylserine. This danger signal activates GPR34 and triggers IL-22 production by ILC3 (89). Deletion of Gpr34 expression in ILC3s impair IL-22 production and tissue repair after gut and skin injury (89).

ILC2s also participate to intestinal homeostasis through the constitutive expression of IL-5 and IL-13. Approximatively 20% of the ILC2s express IL-13 at steady state, and this expression is driven by Tuft cell derived IL-25. The homeostatic role of IL-13 in the gut is unclear, however, it has been shown to control the balance between the type 2 and type 3 immune responses in the skin (90). In absence of IL-25, the number of ILC2s and Tuft cells is reduced, suggesting a reciprocal dependency between these cells at steady state. The Tuft cell-ILC2 circuit can be activated by microbial metabolites. Tuft cells sense succinate synthesized from fermentation of dietary fibers by pathobiont *Tritrichomonas* through its receptor GPR91, thus inducing IL-25 secretion (91). Upon *N. brasiliensis* infection, this feed-forward loop is amplified as IL-13 principally produced by ILC2s during anti-helminth response in turn promotes Tuft cell and goblet cell hyperplasia (92). The “weep-and sweep” effects follow with goblet cell hyperplasia, leading to helminth elimination by massive mucus (weep) production and smooth muscle contraction (sweep) (93). ILC2s also contribute to tissular regeneration after inflammation in the gut with the production of amphiregulin. The alarmin IL-33 derived from damaged epithelial cells stimulates ILC2s to produce the amphiregulin which binds to epidermal growth factor receptor (EGFR) on epithelial cells. The IL-33-amphiregulin-EGFR signaling in epithelium-ILC2 loop promotes epithelial cell proliferation and protects gut from tissue injury and inflammation following DSS-induced colitis (94).

### Diet-mediated control of gut homeostasis

Intestinal ILCs are directly in contact with dietary and microbial metabolites that modulate their functions. Vitamin A and its metabolites and AHR (Aryl Hydrocarbon Receptor) ligands favor ILC3 functions, whereas vitamin D3 and AHR ligands impair ILC3 and ILC2 functions, respectively (95, 96). Cumulative work also highlights the importance of microbiota-derived short chain fatty acids (SCFAs) in modulating ILC functions. SCFAs are metabolites manufactured from bacterial fermentation of dietary fibers. High-fiber diets positively regulate small intestine and colonic ILC3 proliferation, IL-22 secretion, and anti- *C. rodentium* immunity, which is mediated by metabolite-sensing receptors Ffar2 and Ffar3 (97–99). It appears that different SCFAs, including acetate, butyrate, and propionate, would preferentially bind to distinctive SCFA-sensing receptors. For example, butyrate supports intestinal ILC3 functions in Ffar3-dependent and Ffar2-independent manner (97). While SCFAs promote ILC3 expansion and functions, long-chain fatty acids (LCFAs) like palmitic acids are identified to inhibit IL-22 production by ILC3 *in vitro* (100). More research needs to be conducted to elucidate the underlying mechanisms. Furthermore, prostaglandin E2 (PGE2), a lipid derivate, fosters the secretion of IL-22 and HB-EGF *via* targeting its receptors EP4 and EP2, respectively (86, 101). The regulation of the gut homeostasis by dietary cues is extremely complex as a change in the diet can impact not only the immune cells but also Tuft cells, microfold cells or enteroendocrine cells that can indirectly modulate the immune response. Developing new tools to specifically target different metabolic pathways in ILCs will be needed to address the direct regulation of these cells by dietary metabolites and microbial derived metabolites.

### Neuronal relay of gut homeostasis

The maintenance of gut homeostasis heavily relies on neuroimmune interactions, and ILCs appear to be a center of these communications, acting as a controller unit that will instruct effector cells to generate appropriate responses. In addition to the SNS and PNS which originate from the central nervous system, the gastrointestinal tract also possesses its own intrinsic enteric nerve system (ENS) innervating the whole gut. The sympathetic neurons inhibit bowel functions, while parasympathetic neurons activate gut digestion, mobility, and secretion (62). Labeling of cholinergic and adrenergic nerves revealed that each neuronal population spatially occupied distinct layers of the gut. The cholinergic nerves are more abundant in myenteric plexus while the adrenergic nerves dominate in submucosa plexus (102). The ENS composed of both sensory and motor neurons, use neuropeptides such as VIP or neuromedin U (NMU), substance P or CGRP, to modulate the peristaltic motor, absorptive, and secretory functions of the gastrointestinal tract (103). ILCs are anatomically in close proximity to intestinal nerves, suggesting strong neuroimmune interplay between these two partitions (34, 104) (**Figure 3**).

NMU intrinsically promotes ILC2 activation, expansion, and production of type 2 cytokines involving IL-5, IL-9, and IL-13 through NMUR1-G_αq_ signaling. NMU treatment *in vivo* robustly induces anti-*N. brasiliensis* type 2 responses and attenuates gut worm burden, whereas NMUR1^-/-^ mice have compromised worm expulsion. In contrast, α-CGRP plays dual roles in regulating the activation of ILC2s, antagonizing the expansion and IL-13 production of KLRG1^+^ ILC2s but promoting IL-5 secretion (105–107).

At steady state, VIP is released in response to food intake. Intestinal CCR6^+^ILC3 and ILC2 highly express VIP receptor 2 (VIPR2) and VIP expressing enteric neurons project into both the VIPR2^+^ ILC3-enriched cryptopatches and the villi (42, 104, 108). This VIP signaling modulates ILC3 activity in the gut creating a circadian expression of IL-22 in anticipation of pathogenic threats associated with food intake (42). Mechanistically, enteroendocrine cells can secrete cholecystokinin in response to feeding, which rapidly increases the systemic levels of VIP (108). VIP signaling can also synergize with inflammatory signals such as IL-23 and strongly increase the IL-22 expression in ILC3. This synergy increased the resilience of mice to DSS-induced inflammation (42) and to bacterial infection (109). VIP induces the activation of ILC through the increase in intracellular cAMP and calcium levels and it is suggested that VIP potentiates ILC function by increasing glycolysis-based energy metabolism (108).

Interestingly, Talbot et al. reported a negative effect of VIP on IL-22 production correlated with exacerbated intestinal bacterial infection (104). In this study, VIP fosters lipids absorption at the expense of pathogen protection. Even if the cause of the discrepancies between these studies are unclear, it is not surprising that the same neuropeptide can have different physiological outcomes, depending on the animal facility, the activation status, the proportion of ILC3 subsets, or the presence of other environmental cues that differentially synergize or antagonize the VIP signaling.

The role of the PNS and SNS on ILC regulation is less understood. Similar to lung ILC2s, sympathetic nerves appear to inhibit intestinal ILC2 proliferation and functions in producing IL-5 and IL-13 after *N. brasiliensis* infection (34). ACh has been shown to support regenerative capacity of peritoneal ILC3 by inducing PCTR1 (protectin conjugates in tissue regeneration 1), placing the ILC3 at the center of resolution circuitry controlled by the central nervous system (110). PCTR1 is a pro-resolving mediator that exerts potent tissue regenerative effects by enhancing macrophage recruitment and phagocytosis and reducing leukocyte infiltration and inflammation. Consequently, vagotomy reduces peritoneal ILC3 numbers and delays the resolution of infection-induced inflammation (110). Finally, enteric glial cells also prove to orchestrate ILC3 functions by direct GDNF-RET signaling pathway. RET deficiency in ILC3 results in diminished IL-22 production and aggravates intestinal inflammation and injury following *C. rodentium* infection or DSS-induced colitis (111).

The neuroimmune interactions that occur in the gut are intense but vary with time and inflammation. These interactions are not simple to study and spatiotemporal analysis would be needed to better understand the consequences of these interactions on the function of the tissue. The expression of the neuropeptide receptors is not homogeneous on the different ILC subsets. This could indicate that specialized groups of ILCs, that are likely localized at different strategic locations, are sensitive to specific neurotransmitters. This could lead us to rethinking the classifications of ILC subsets. Instead of using immunological markers such as KLRG-1 or NKp46 to define ILC2 and ILC3 subsets respectively; we may define subsets based on the expression of the receptor for host derived modulators. Comparing the activity of the VIPR2^+^ ILC3 and VIPR2^-^ could help to better understand the homeostatic function of these cells. Understanding the functional differences between nicotinic and muscarinic receptors expressing ILC2 could reveal the molecular network promoting the pro- and anti-inflammatory property of these cells.

## Perspectives

The study of tissue-resident immune cells, ILCs in particular, has highlighted how constitutive activities of immune cells contribute to tissue functions rather than being limited to the immune surveillance and pathogen elimination. This feature could be one of the key differences of ILCs compared to conventional T cells. Although they share common transcriptional regulators with T cells, the tissue residency of ILCs and their capacity to integrate physiological host-derived signals at steady state confers a unique place for ILCs in the immune system. The studies cited in this review show that ILC, not only, respond to different physiological cues but also actively produce neuropeptides such as CGRP (58), ACh (53, 54), serotonin (112) or methionine-enkephalin (24). The homeostatic functions of ILCs, control the balance between the type 1, 2 and 3 response but also modulate non-immunological function such as thermal regulation, lipid absorption and storage, neuropeptide production and tissue repair extending the capacity of these cells well beyond the canonical function of immune cells. Immunologists will have new horizons to explorer that will require multidisciplinary approaches to understand how ILCs, and tissue resident immune cells in general, modulate physiological functions.

Physiological modulators are dynamically regulated during inflammation allowing a negative feedback loop to control the inflammatory responses and promote tissular protection. What is more interesting is that these modulators are also expressed to maintain tissue homeostasis, in absence of harmful pathogens. A dysregulation, or a disruption in the sensing of physiological cues can lead to tissue inflammation by modifying the constitutive activity of ILCs. For example, unbalanced type 1 and type 2 immune responses in the adipose tissue led to metabolic disorder, or the disruption of type 3 response in the gut provoked dysbiosis.

The increasing prevalence of chronic inflammatory diseases in the most developed countries are associated with fat-rich diets, disrupted circadian rhythms, and chronic stress. Chronic inflammatory diseases have common pathogenic features in dysregulated host immune responses. There are opportunities here to improve our understanding about the impact of physiological cues on the immune system and explore new therapeutic strategies to re-establish homeostasis to treat these conditions. Due to their chronic nature, inflammatory conditions are linked to metabolic perturbation, and higher risk of developing chronic diseases including type 2 diabetes and cancer. Understanding how physiological signals modulate immune responses could not only reveal the mechanisms that underlie the development of sterile inflammation but also provide new insights into the pathogenesis of comorbidities that have a major impact on human health.

## Author contributions

All authors contributed to the manuscript and read, edited, and approved the final manuscript.

## Funding

LX is supported by a PhD scholarship from the University of Melbourne. This work was supported by grants and fellowships from the National Health and Medical Research Council (NHMRC) of Australia (CS 1165443; SN 1155342), Australian Research Emerging Leader Fellowship (C.S 2008090). This work was made possible through Victorian State Government Operational Infrastructure Support and Australian Government NHMRC IRIIS.

## Conflict of interest

The authors declare that the research was conducted in the absence of any commercial or financial relationships that could be construed as a potential conflict of interest.

## Publisher’s note

All claims expressed in this article are solely those of the authors and do not necessarily represent those of their affiliated organizations, or those of the publisher, the editors and the reviewers. Any product that may be evaluated in this article, or claim that may be made by its manufacturer, is not guaranteed or endorsed by the publisher.
